# Virus usurps alternative splicing to clear the decks for infection

**DOI:** 10.1186/s12985-023-02098-9

**Published:** 2023-06-20

**Authors:** Ruixue Li, Shenyan Gao, Huayuan Chen, Xiaozhan Zhang, Xia Yang, Jun Zhao, Zeng Wang

**Affiliations:** 1 College of Veterinary Medicine, Henan Agricultural University, Zhengzhou, People’s Republic of China; 2 College of Veterinary Medicine, Henan University of Animal Husbandry and Economy, Zhengzhou, People’s Republic of China

**Keywords:** Alternative splicing, Virus infection, Viral protein expression, Immune evasion

## Abstract

Since invasion, there will be a tug-of-war between host and virus to scramble cellular resources, for either restraining or facilitating infection. Alternative splicing (AS) is a conserved and critical mechanism of processing pre-mRNA into mRNAs to increase protein diversity in eukaryotes. Notably, this kind of post-transcriptional regulatory mechanism has gained appreciation since it is widely involved in virus infection. Here, we highlight the important roles of AS in regulating viral protein expression and how virus in turn hijacks AS to antagonize host immune response. This review will widen the understandings of host-virus interactions, be meaningful to innovatively elucidate viral pathogenesis, and provide novel targets for developing antiviral drugs in the future.

## Background

Virus takes full advantage of cellular machinery to sustain themselves and promote replication. AS, a conserved and critical mechanism of processing pre-mRNA into mRNAs in eukaryotes, has been reported to deeply involved in multiple biological processes. Many studies demonstrate that AS is at the mercy of virus to positively regulate its infection. Here, we briefly highlight how the virus hijacks AS to diversity its proteome and counteracts host immune response. This review widens the mechanisms of host-virus interactions and provides a promising target for the development of novel strategies to antagonize virus infection.

## Introduction

It’s well known that the quantity of proteins in eukaryotes is far outnumbering the protein-encoding messenger RNAs (mRNAs) due to AS, making it possible to expand functional proteome with limited genes. Precursor messenger RNA (pre-mRNA) splicing, dating back to 1978 [1, 2], is a critical step in post-transcriptional regulation by removing introns and ligating exons to form mature mRNAs, which contributes to the increased organismal complexity [3]. Genome-wide analysis shows that approximately 90–95% of human genes undergo AS, generating proteins with similar, dissimilar, and even mutually exclusive functions [4].

AS takes pre-mRNA as the primary template to generate various mRNA transcripts. Mechanistically, AS is sophisticatedly regulated by *cis*-acting elements, such as exonic/intronic splicing enhancers/silencers (ESE/S, ISE/S), and *trans*-acting factors, such as serine arginine-rich (SR) proteins, heterogeneous nuclear ribonucleoproteins (hnRNPs), and RNA secondary structure [5–7]. Recognizing 5’ and 3’ splice site and differentiating exons from introns by spliceosome are crucial for efficient and accurate splicing [8]. The spliceosome is a large RNA-protein complex that consists of five small nuclear ribonucleoprotein particles (U1, U2, U4/U6, and U5 snRNPs) and hundreds of non-snRNP factors [9, 10]. Generally, the exons and introns could be precisely excised when the boundaries are clearly outlined. However, if the exon–intron definition is blurred by negative regulation, the intron might be partially or integrally preserved, generating different transcripts from the wildtype one [11]. AS events have been classified into five main types at least, including the exon skipping, intron retention, alternative 3’ splice site, alternative 5’ splice site, and mutually exclusive exons [12, 13]. Not surprisingly, AS is a key technique for increasing transcriptome and proteomic diversity from a small genome, since more than one splicing type could be simultaneously observed in a single transcript isoform [14].

Functionally, AS events are deeply involved in many biological and pathological processes, such as sex determination [15], organ development [16], cancer occurrence [17], and even virus infection [18]. Based on the facts that virus unscrupulously disrupts cellular pathways and splicing process is a ubiquitous regulatory mechanism of gene expression, AS is undoubtedly deeply involved in virus infection. Therefore, this review underlines the inseparable relation between virus and AS, especially how virus fully utilizing AS to work for itself, and provides a promising prospect for illustrating pathogenesis and developing antiviral drugs.

## Virus hijacks cellular splicing machinery to promote infection

After infection, virus takes over the cellular pathways to sustain themselves in the host cells. Since AS plays large part in shaping the transcriptome, it is no doubt that virus evolves to selfishly re-wire the AS response for its own benefit.

### AS is critical to diversify the viral proteome

Viruses are nothing but small protein capsules enclosing the simple genetic material. For certain ones, the splicing machinery is hijacked to produce viral proteins and maintain the lifecycle of the virus. Human immunodeficiency virus type 1 (HIV-1), the etiologic agent of acquired immunodeficiency disease syndrome (AIDS), has always been the subject to study the important role of AS in its life cycle. HIV-I usurps host splicing mechanism to generate over 40 different spliced mRNA from a single full-length unspliced primary transcript, which are further translated into diverse products including structural proteins and regulatory factors [19]. The sophisticated process depends on the cooperation of multiple positive and negative factors, such as *cis*-regulatory elements in HIV-1 RNA and *trans*-acting cellular and viral proteins. Over the past several decades, considerable progress has been made in understanding the mechanisms of how HIV-1 regulates its RNA splicing. Since high mutation frequency of HIV-1 RNA resulting in drug resistance, antiviral strategies targeting HIV-1 splicing has become the promising therapy to curb AIDS [20]. Apart from HIV-1, many other viruses depend on AS to complete its lifecycle. Here, we take some typical viruses as examples to show how the AS is usurped to maximize the coding potential of viral genome (Table 1).

**Table 1:** Types of AS have been identified in AdV, HPV, and IAV

Virus	Viral gene	Splice type	Regulator factor	Consequence
AdV	E1A RNA	Alternative 5’ splice site and alternative 3' splice site	Celluar SR proteins	Generate 9S, 10S, 11S, 12S, and 13S viral mRNA
	L1 RNA	Alternative 3’ splice site	Cellular SR proteins and viral E4-ORF; viral *cis*-acting elements, 3RE and 3VDE	Generate 52/55 K and IIIa viral mRNA
HPV	E6/E7 RNA	Alternative 3’ splice site	Cellular hnRNP and SR proteins; viral *cis*-acting elements SA409	Generate E6 and E7 mRNA
IAV	M RNA	Alternative 5’ splice site	Cellular SR proteins, hnRNP protein family and NS1-BP, and viral polymerase complex as well as NS1	Generate M2 mRNA
	NS RNA	Alternative 5’ splice site	Viral NS1	Generate NS2 mRNA

#### Adenovirus

Adenovirus (AdV) genome is compact with rarely redundant nucleotides that are not transcribed or serve regulatory functions. Generally, it could be divided into different transcription units based on their expressive phase: the early (E1-E4) and the late genes (L1-L5) [21]. Thereinto, AdV E1 pre-mRNA has been well-known to undergo splicing by using five 5’ splice sites and one common 3’ splice site to excise introns from 1112 to 1225 nucleotides (nt), from 974 to 1225 nt, from 637 to 852 nt and 1112 to 1225 nt, from 637 to 852 nt and 974 to 1225 nt, or from 637 to 1225 nt, respectively, to generate 13S, 12S, 11S, 10S, or 9S mRNA, respectively [22, 23]. During lytic infection, 13S and 9S forms are the most abundant in early and late phases, respectively, and the shift from 13 to 9S is dependent on the SR splicing factors in an appropriate ionic condition [24, 25]. Notably, AS of E1A pre-mRNA is highly sensitive to changes in various parameters, therefore, it has successfully been used as a model substrate to characterize the function of SR proteins [26]. Studies have demonstrated that ASF/SF2 (especially its second RNA binding domain) and SC35 enhance proximal 13S mRNA splicing [27, 28], SRp20 enhances 12S mRNA splicing [29], and SRp54 enhances 9S mRNA splicing [30]. The distinct trans-acting properties of SR proteins might due to their different binding ability between arginine/serine-rich domains with U1 snRNP [31].

Besides early genes, the adenoviral major late transcription unit (MLTU) is sophisticatedly manipulated by AS to generate approximately 20 mRNAs. The MLTU produces a primary transcript of ~ 28,000 nt, which becomes polyadenylated at one of five positions (L1-L5 mRNA families) with co-terminal 3’-ends. Thereinto, L1 is an alternative spliced gene where the last intron is spliced using a common 5’ splice site and two competing 3’ splice sites (11,040 nt and 12,308 nt) to generate two cytoplasmic mRNAs, the 52/55 K and the IIIa, respectively [32, 33]. 52/55 K is indispensable for viral genome encapsulation [34], and IIIa protein serves its best characterized function as a structural protein in the capsid [35]. Intriguingly, proximal 3’ splice site located at 11,040 nt is activated in the early phase of infection, resulting in an exclusive production of 52/55 K. However, the distal 3’ splice site mapped at 12,308 nt becomes active to generate almost equal amount of 52/55 K and IIIa in the late time [36, 37]. Further study found that IIIa splicing is tightly controlled by two *cis*-acting viral elements, the 49 nt IIIa repressor element (3RE) and the 28 nt IIIa virus-infection dependent splicing enhancer (3VDE). The 3RE binds the hyper-phosphorylated form of SR proteins to inhibit the spliceosome assembly on the IIIa 3’ splice site [38, 39], therefore blocking IIIa expression in the early infectious stage. This inhibition is released by viral E4-ORF4 through inducing SR proteins dephosphorylation in order to recruit U2 snRNP binding the branch point [40]. The other element 3VDE, consisting of the IIIa branch point sequence, pyrimidine tract, and AG dinucleotide, is necessary to activate IIIa splicing in the AdV-infected HeLa-NE. Although 3VDE takes effect through an U2AF-independent manner, the L4-33 K has been identified as an AdV-encoded alternative RNA splicing factor to active IIIa expression [41, 42]. These results indicate that virus could not only “steal” but plant “inside man” within splicing machinery to regulate viral protein expression.

Apart from the regulatory factors mentioned above, notably, RNA modification and dsRNA production play pivotal roles in efficient splicing of AdV RNAs. N6-methyladenosine (m^6^A), the most prevalent modification in cellular RNAs, has been found in early and late adenoviral transcripts [43]. Depletion of m^6^A writer methyltransferase like 3 (METTL3) specifically impacts viral late transcripts by reducing their splicing efficiency, and this biased-effect could be extended to all the multiply spliced AdV late RNAs [44]. Moreover, AdV mutants lacking virus-directed ubiquitin ligase activity, but not wildtype ones, produce abundant dsRNA within the nucleus of infected cells, leading to form intron/exon base pairs between top and bottom strand transcripts. Consequently, cytoplasmic dsRNA sensor PKR is translocated to the nucleus, igniting host innate immune response and blocking AS of viral RNAs [45]. Therefore, m^6^A modification and preventing dsRNA formation are necessary for avoiding restriction by host immune sensors and promoting efficient splicing of viral RNAs.

#### Human papillomavirus

Similarly, Human papillomavirus (HPV) genome could be divided into exclusively early genes (E6 and E7), early and late genes (E1, E2, E4 and E5) and exclusively late genes (L1 and L2). Transcriptions from promoter p97 and p670 generate pre-mRNAs encoding all the early and late genes, respectively [46]. Subsequently, the 5’ and 3’ splice sites are directly recognized by splicing factors, such as hnRNP or SR proteins, to either repress or stimulate the use of a specific splice site, which starting the splicing procedure to produce early and late proteins [47, 48]. For instance, E2 inactivates early polyadenylation signal pAE, causing a switch from early to late gene expression [49]. A splicing enhancer on E2 mRNA interacts with amino acids 236–286 of cellular RNA binding protein hnRNP G, contributing to specific splicing at the 3’ splice site SA2709 to generate E2 protein [50].

Besides E2, AS of E6 and E7 must be mentioned since the increased expression of the two oncoproteins strongly facilitate HPV-associated tumorigenesis [51, 52]. E6 and E7 target p53 and pRB, respectively, to inactivate tumor suppressors through proteasome-mediated degradation [53]. Notably, E6 and E7 are derived from the same polycistronic transcript, which contains three exons and two introns with three 3’ splice sites in intron 1. AS of intron 1 leads to produce four different alternative spliced mRNAs, E6 full length (E6fl), E6*I, E6*II, and E6*X (also called E6^E7) [54]. The three putative E6* proteins share the same N-terminal 44 amino acids of E6fl, with the C-terminal truncations or frame shifts into the E7 open reading frame [55]. Thereinto, E6*I, the most abundant isomer in HPV-related cancers, has been suggested to encode E7 [56–58]. E6/E7 splicing is precisely regulated by the interaction of *cis*-acting elements, including branch point sequence (BPS) and splicing silencers, and *trans*-acting factors. Several consecutive nucleotides located within the E6-coding region upstream of 3’ splice site SA409, such as AACAAAC for HPV16 and AACUAAC for HPV18, have been identified to be the BPS, which are closely related with the efficiency of E6*I splicing and further affecting the production of E7 [59]. The crucial point mutation could interrupt BPS binding activity to U2 snRNP, causing inefficient splicing to produce E7 protein. Additionally, splicing silencers have been mapped to interact with hnRNP A1/A2, thereby reducing the expression of E6*I and E7 [60, 61]. Other *trans*-acting factors, such as hnRNP G and SRSF2, could also negatively disrupt the balance of E6/E7 proportion and further cause apoptosis of infected cells [50, 62]. Since E6/E7 is important for HPV tumorigenicity, regulating AS to manipulate their expression might be the promising therapy to antagonize viral carcinogenesis.

#### Influenza virus

In addition to DNA viruses, RNA ones have been reported to usurp host splicing mechanism to expand the coding capacity of their limited genes [63]. The genome of influenza virus (IAV) consists of eight negative-sense RNA segments, and both M and NS genes are well-known to express different spliced transcripts. There are four differentially spliced isoforms from segment 7, M1, M2, M3, and M4. M1 and M2 are essential for viral nuclear export, virion packaging, and progeny budding [64, 65], and while no known function has been found for M3 and M4 [66]. M42, an M2-related protein, is expressed from M4 mRNA utilizing an alternative start codon and is hypothesized to be a novel ion channel protein to replace the function of M2 [67]. Shih et al*.* reports that viral polymerase complex and cellular splicing factor SF2/ASF jointly regulate the utilization of alternative 5’ splice sites in M pre-mRNA and control the M2 expression during infection [68, 69].Other study finds that cellular hnRNP K and NS1-BP proteins direct M segment splicing through binding 5’ splice site of M2 mRNA. Mutation of either or both the hnRNP K and NS1-BP-binding sites results in M segment mis-splicing and attenuated IAV replication [70]. Liu et al*.* further identifies another cellular factor SRSF5 directly involves in M2 production. SRSF5 binds crucial sites 163/709/712 in M pre-mRNA via its RRM2 domain, and recruits U1 snRNP through interacting with U1A to increase M2 expression, subsequently enhancing virus replication in A549 cells and pathogenicity in mice [71]. Apart from polymerase complex and cellular splicing factors, NS1 has been demonstrated to participate in M2 expression [72, 73]. Although deleting NS1 gene (DelNS1) usually leads to severe attenuation of IAV in interferon-competent cells, A14U, an adaptive mutation in the 3’ noncoding region of M segment could compensate the replication of DelNS1 through restore M2 expression [74]. This data suggests that NS1 is involved in IAV replication through modulating the splicing process of M transcripts. Intriguingly, Calderon et al*.* shows that avian IAV M segment is prone to enhancing splicing efficiency to produce excessive M2 protein when transcribed in mammalian cells. The aberrant high levels of M2 proton channel prevent fusion between autophagic vesicles with lysosomes, which in turn reducing the efficiency of viral replication and limiting the zoonotic potential of avian IAVs [75]. This data is the auxiliary evidence for species barrier of avian IAV, however, the exact role of mammalian IAV M2 in host adaptation still needs to be further studied.

The splicing of segment 8 creates mRNAs that encode nonstructural protein 1 (NS1), NS2, and NS3. The full length NS1 is an RNA-binding protein, which is essential for efficient IAV replication and virulence due to its roles in counteracting host immune response and regulating viral protein expression [76]. Two different 5’ splice sites are used to generate truncated NS2 and NS3 [77], which also play important roles in virus lifecycle, such as NS2 facilitating virus budding and antagonizing the production of interferon (IFN) [78, 79], and NS3 stimulating cytokines to promote pathogenicity [80]. Since NS1 plays critical roles in the splicing of viral genes, it has been reported to block the splicing and nucleocytoplasmic transport of its own mRNA, but not others, through N-terminal region in a transient replication/transcription system, suggesting that NS1 might maximize its function through suppressing the splicing rate [81–84].

### Virus alters cellular genes splicing to clear the decks for infection

AS is significantly important for every aspect of human biology, not surprisingly, virus infection could massively disrupt AS to pave the way for effective infection.

#### Virus infection cause global host AS changes

Most nuclear-encoded genes in eukaryotes are modified by AS, which could be targeted by virus for their own advantage. To date, many viruses, such as herpesvirus (HSV) [85], dengue virus (DENV) [86], enterovirus 71 (EV71) [87], zika virus (ZIKV) [88], severe acute respiratory syndrome coronavirus 2 (SARS-CoV-2) [89], and IAV [90], have been reported to change the global AS patterns in infected cells. And viruses alter cellular genes splicing mainly through the following ways:

1. *Virus regulates the expression of splicing factors* Human cytomegalovirus (HCMV) infection upregulates the expression of cellular RNA-binding protein CPEB1, resulting in a global shortening of 3’ untranslated regions and lengthening of poly(A)-tails [91]. The μ2 protein encoded by mammalian orthoreovirus (MRV) M1 gene could reduce the protein level of U5 snRNP core components [92], which is well-known to be important in recognition and subsequent removal of the intron [93]. As RNA-binding proteins, U5 snRNP components are mainly located in nucleus, while MRV replicates in cytoplasm, the battle field in which μ2 protein affects either the stability or degradation of the unassembled U5 proteins before their nuclear import.
2. *Virus changes the subcellular localization of splicing factors* M1, M2, and M3 proteins of vesicular stomatitis virus (VSV) induce a cytoplasmic relocation of hnRNPs to inhibit host genes transcription [94, 95]. Similarly, seneca valley virus (SVV) infection causes cytoplasmic retention of hnRNP K, which is subsequently cleaved and degraded by viral 3C^pro^, to promote virus infection but inhibit cellular gene expression [96]. Human rhinovirus 16 (HRV16) 2A protease directs the splicing factor SRp20 relocating from nucleus to cytoplasm in HeLa cells as early as 2 h post infection, in order to initiate internal ribosome entry site (IRES)-mediated viral gene translation [97].
3. *Virus interacts with the splicing machinery to alter splice site choice* DENV NS5 reduces splicing efficiency of endogenous mRNAs through binding U5 snRNP core components CD2BP2 and DDX23 to increase the ratio of intron retention, rendering an advantageous cellular environment for DENV replication [86]. The fingers domain of EV71 3D^pol^, an RNA-dependent RNA polymerase (RdRp), targets the C-terminal region of pre-mRNA processing factor 8 (Prp8) to inhibit the second step of the splicing process, resulting in accumulation of the lariat form and a reduction on mRNA levels [98]. SARS-CoV-2 Nsp16 protein binds the mRNA recognition domains of U1/U2 spliceosomal components and acts to suppress global mRNA splicing upon infection [99].
4. *Virus non-coding RNAs sponge off splicing factors* As important emerging and reemerging human pathogens, flavivirus infections produce viral noncoding RNAs, known as sfRNAs, involved in viral replication and pathogenesis [100, 101]. ZIKV has been reported to cause neuropathology via disturbing the splicing process of cellular transcripts [102], and sfRNAs deserve all the credit. sfRNAs from ZIKV could serve as a sponge for over 20 RNA-binding proteins to disturb post-transcriptional modification in cells, including splicing, RNA stability, and translation. As a component of U2 snRNP, for example, SF3B1 strongly restricts viral infection, while sfRNAs dysregulates its function to promote ZIKV propagation [103].

Although many viruses regulate splicing factors via different ways, notably, it could not directly demonstrate that these pathogens exert strong influence on global host AS. Since numerous splicing factors co-regulate host genes splicing events, which one(s) of them are altered by virus infection still need further illustration.

#### Virus utilizes negative splicing variants of innate immune response genes to inhibit antiviral defense

**Fig. 1:**
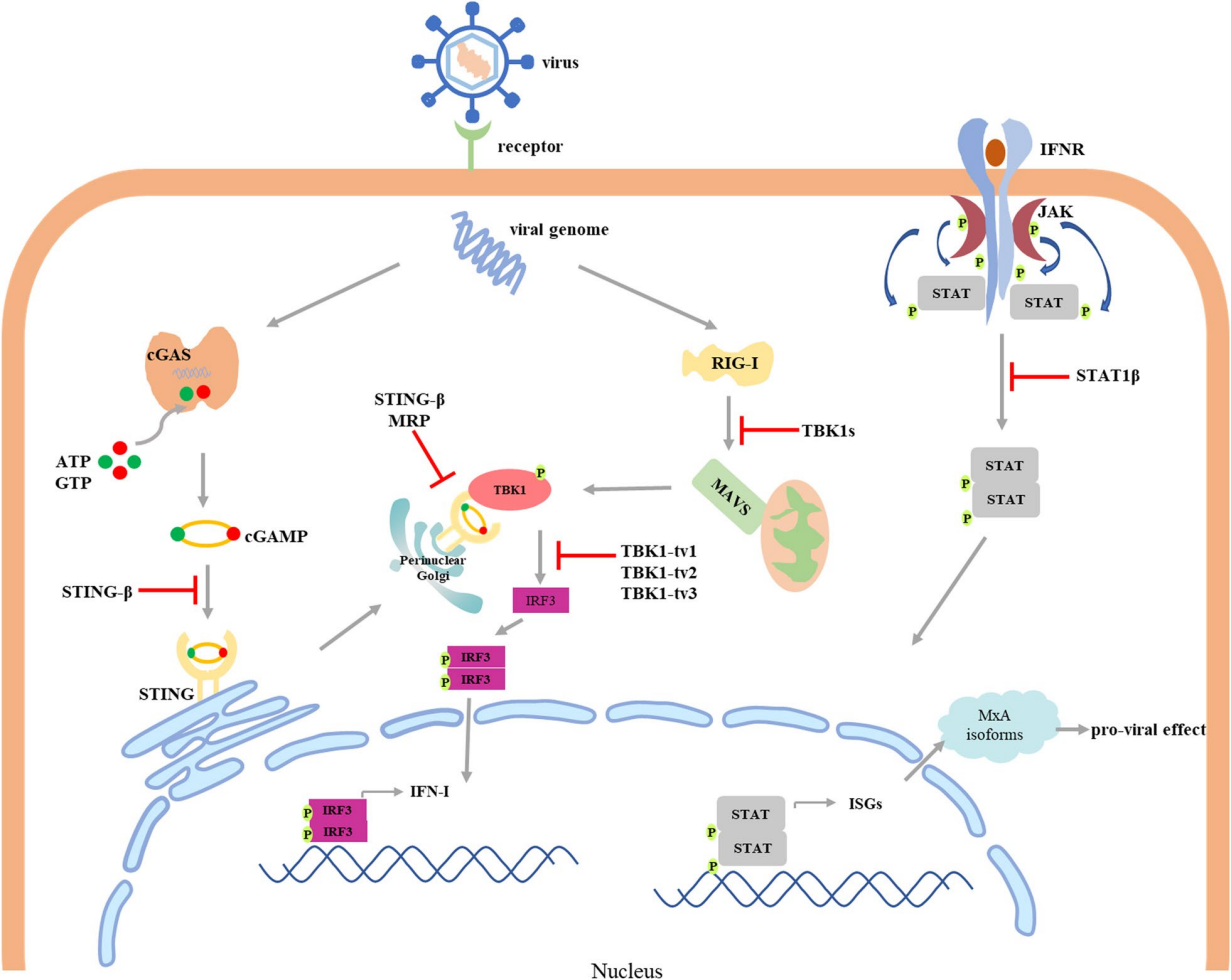
Virus manipulates the splicing of specific checkpoints in innate immune pathways to evade antiviral defense. STING-β negatively regulates the signal transduction through inhibiting cGAMP-STING and STING-TBK1 interactions and facilitates VSV-GFP and HSV-GFP replication. MRP similarly promotes SeV and VSV propagation through blocking STING-TBK1 interaction to inhibit IFN-I response. TBK1s binds to RIG-I to inhibit the interaction between RIG-I and MAVS upon SeV infection. TBK1s targets RIG-I to inhibit interaction between RIG-I and MAVS upon SeV infection. In SVCV-infected epithelioma papulosum cyprini cells, TBK1_tv1, TBK1_tv2, and TBK1-tv3 competitively associate with TBK1 and IRF3 to inhibit the formation of TBK1-IRF3 complex. And TBK1-tv3 additionally promotes the degradation of TBK1 and IRF3 through the ubiquitin–proteasome pathway and the lysosomal pathway, respectively. EBV SM protein upregulates the expression of STAT1β to exert negative effect on IFN response. HSV-1 and IAV creates a pro-viral state in infected cells by promoting the production of MxA isoforms

The innate immune system is the first line of defense against invading pathogens. The pathogen-associated molecular patterns (PAMPs) are instantly recognized by pattern recognition receptors (PRRs) to ignite an array of anti-microbial immune responses via the induction of various chemokines, pre-inflammatory cytokines, and IFNs. To date, many negative splicing transcripts of immune molecules have been found in humans, mouse, Chinese tree shrews, and zebrafish [104]. Which are at the behest of virus to promote infection. Here, we take stimulator of interferon genes (STING), TANK binding kinase 1 (TBK1), and interferon stimulated gene (ISG) as examples to illustrate how virus exploits their negative splicing variants to antagonize host antiviral defense (Fig. 1).

##### Splicing variants of STING negatively regulate IFN-I production to promote virus infection

STING is an endoplasmic reticulum membrane dimeric protein that contains transmembrane domain (TMD) in the N-terminus and an intracellular soluble portion in the C-terminal domain (CTD) [105]. The N-terminal region regulates its cellular localization and homodimerization [106], and the CTD functionally docks downstream molecules, including TBK1/IKKε and IRF3/IRF7, to transmit signal [107]. Besides the full length one, four kinds of STING splicing variants, MRP, isoform2, isoform3, and STING-β, have been identified in human cells. Since all the former three are absent for exon 7 compared to the canonical mRNA, they are unable to interact with TBK1 and interferon regulatory factor 3 (IRF3) due to lacking CTD. STING-β, transcribed by using an alternative first exon and an alternative 5′ transcription initiation site, contains the functional CTD, but without TMD. Due to lacking important function domain, the four splicing variants are always usurped by virus to counteract antiviral response. STING-β has been identified to bind cyclic GMP-AMP (cGAMP), STING, and TBK1 to antagonize IFN expression. Therefore, IFN susceptible VSV-GFP and HSV-GFP replication is significantly attenuated in HEK293 cells incubated with conditioned media from STING-β knockdown THP-1 cells [108]. Chen et al. demonstrates that overexpression of MRP inhibited STING-mediated activation of IFN-β promoter by disrupting the STING-TBK1 interaction. MRP could even promote RNA virus replication, such as sendai virus (SeV) and VSV, via negatively regulating the induction of IFN signaling pathways [109]. Moreover, recent study finds that RNA-binding protein LUC7L2 downregulates STING level by mediating its intron 3 retention, leading to attenuate the innate immune responses to HSV-1 infection [110]. Notably, the function of the same isomer might be discrepant during different virus infection, and the exact molecular mechanism still needs to be further illustrated.

##### Splicing isoforms of TBK1 downregulate the expression of virus-triggered IFN-I

TBK1 is an important signaling hub downstream of RIG-I like receptors (RLRs) and DNA-sensing receptors (DSRs). Similarly, it is subject to AS and produce isomer TBK1s, which lacks kinase domain since missing exons 3–6 compared to the full-length mRNA. Upon SeV infection, TBK1s is upregulated in human and mouse cells, and targets caspase recruitment domain (CARD) of RIG-I, but not TBK1, to disrupt interaction between RIG-I and mitochondrial antiviral signaling (MAVS), resulting in decreasing the production of IFN-β [111]. Additionally, similar spliced isoforms, TBK1-tv1, TBK1-tv2 and TBK1-tv3, have been identified in zebrafish, and both the three act as negative regulators in RIG-I/MAVS/TBK1/IRF3 axis during spring viremia of carp virus (SVCV) infection. However, the underlying mechanism is different from that of TBK1s. TBK1_tv1 and TBK1_tv2 competitively associate with TBK1 and IRF3 to inhibit the formation of TBK1-IRF3 complex, impeding the phosphorylation of IRF3 mediated by TBK1 [112]. And TBK1-tv3 also promotes the degradation of TBK1 and IRF3 through the ubiquitin–proteasome pathway and the lysosomal pathway, respectively [113]. These data suggest that different splicing variants generated from the same gene could achieve the same end through different ways.

##### Virus induces aberrant splicing of ISGs to counteract IFN-mediated host immune response

IFN interacts with cell surface receptors and initiates a signaling cascade through JAK-STAT pathway, leading to the expression of numerous ISGs involved in the innate immune system response [114]. STAT1, an integral mediator of both IFN-I and IFN-II signal transduction pathways, contains two isoforms, STAT1α and STAT1β. Since generated by splicing in the last intron, STAT1β lacks the *trans*-acting domain and is inactive in signal transduction [115]. EBV SM protein is an RNA-binding protein in the lytic phase and acts as a *trans*-acting factor to regulate viral RNA splicing [116]. Importantly, SM has been reported to exert negative effect on IFN response via disproportionately increasing the abundance of STAT1β [117].

Myxovirus resistance protein A (MxA), a classical ISG induced by IFN-I, plays antiviral role in various virus infections. In humans, MxA gene contains 17 exons and the encoded protein consists of N-terminal GTPase domain and C-terminal central interactive domain and leucine zipper domain [118]. A variant MxA isoform (varMxA) with conserved GTPase domain and a novel CTD due to the deletion of 14–16 exons is induced in HSV-1 infected cells. Unlike MxA with vibrant antiviral activity, varMxA favors HSV-1 replication and enhances the production of infectious virus progeny [119]. Shu et al. reports that compared to healthy controls, patients infected with avian H7N9 IAV carry higher rate of heterozygous single-nucleotide variants in MxA gene, some of which might affect the effective and accurate splicing to generate MxA protein. Importantly, nearly all the inactive MxA variants exert a dominant-negative effect on the antiviral function of wild-type MxA, suggesting that variations in MxA gene are closely correlated with susceptibility to IAV [120]. Notably, whether and how varMxA and other isoform counterparts directly inhibiting the antagonism of MxA on virus infection still needs to be further studied.

## Conclusions

AS is a mechanism that allows single gene to generate multiple transcripts that encode proteins with diverse and even antagonistic functions. It is ubiquitous for virus to usurp splicing mechanism to regulate viral protein expression and create a pro-viral environment. Extensive studies have demonstrated that virus caused aberrant splicing of cellular genes significantly contributes to pathogenicity in infected cells. Identifying anomalous isoforms induced by virus and studying their functions could be the biomarker for diagnosis and the primary target for novel antiviral strategies. Since splicing isoforms of the same gene are divergent in different model animals, however, their formation and functional mechanism need to be rigorously investigated before clinical trial.

## Data Availability

All data generated or analyzed during this study are included in this published article.

## Abbreviations

AS: Alternative splicing
ESE: Exonic splicing enhancers
ESS: Exonic splicing silencers
ISE: Intronic splicing enhancers
ISS: Intronic splicing silencers
SR: Serine arginine-rich
hnRNPs: Heterogeneous nuclear ribonucleoproteins
AdV: Adenovirus
MLTU: Major late transcription unit
3RE: IIIa repressor element
3VDE: IIIa virus-infection dependent splicing enhancer
HPV: Human papillomavirus
IAV: Influenza virus
IFN-I: Type I interferon
HSV: Herpesvirus
DENV: Dengue virus
ZIKV: Zika virus
EV71: Enterovirus 71
SARS-CoV-2: Severe acute respiratory syndrome coronavirus 2
HCMV: Human cytomegalovirus
MRV: Mammalian orthoreovirus
VSV: Vesicular stomatitis virus
SVV: Seneca valley virus
HRV16: Human rhinovirus 16
IRES: Internal ribosome entry site
RdRp: RNA-dependent RNA polymerase
PAMPs: Pathogen-associated molecular patterns
PRRs: Pattern recognition receptors
STING: Stimulator of interferon genes
TBK1: TANK binding kinase 1
ISG: Interferon stimulated gene
TMD: Transmembrane domain
CTD: C-terminal domain
IRF3: Interferon regulatory factor 3
cGAMP: Cyclic GMP-AMP
SeV: Sendai virus
RLRs: RIG-I like receptors
DSRs: DNA-sensing receptors
MAVS: Mitochondrial antiviral signaling
SVCV: Spring viremia of carp virus
MxA: Myxovirus resistance protein A
